# Biomimetic Antigravity Water Transport and Remote Harvesting Powered by Sunlight

**DOI:** 10.1002/gch2.202000043

**Published:** 2020-09-06

**Authors:** Hongya Geng, Cunjing Lv, Mingmao Wu, Hongyun Ma, Huhu Cheng, Chun Li, Jiayin Yuan, Liangti Qu

**Affiliations:** ^1^ Department of Chemistry Tsinghua University Beijing 100084 P. R. China; ^2^ Applied Mechanics Laboratory Department of Engineering Mechanics and Center for Nano and Micro Mechanics Tsinghua University Beijing 100084 P. R. China; ^3^ State Key Laboratory of Tribology and Key Laboratory for Advanced Materials Processing Technology Department of Mechanical Engineering Tsinghua University Beijing 100084 P. R. China; ^4^ Department of Materials and Environmental Chemistry Stockholm University Stockholm SE‐106 91 Sweden

**Keywords:** antigravity water transport, artificial trees, biomimics, remote water harvesting, sunlight

## Abstract

Antigravity water transport plays important roles in various applications ranging from agriculture, industry, and environmental engineering. In natural trees, ubiquitous water‐flow over 100 m high from roots through the hierarchical xylem to leaves is driven by sunlight‐powered continuous evaporation and the negative pressure. Inspired by natural trees, herein an artificial trunk‐leaf system is built up to structurally mimic natural trees for a continuous antigravity water delivery. The artificial tree consists of directional microchannels for antigravity water transport, and a top leaf‐like hybrid hydrogel that are acts as continuous solar steam evaporator, plus a purposely engineered steam collector. It is found that continuous uniform microchannels of acetylated chitin optimize and enhance capillary rise (≈37 cm at 300 min) and reduce vertical water transport resistance. A remote water harvesting, and purification is performed with a high rate of 1.6 kg m^−2^ h^−1^ and 184 cm in height under 1 sun irradiation and the collection efficiency up to 100% by evaporative cooling technique. It is envisioned that the basic design principles underlying the artificial tree can be used to transform solar energy into potential energy.

## Graphical abstract

Antigravity water transport is necessary for industry, agriculture, environmental engineering, and daily life, which consumes electricity or other forms of fossil‐based energy. Inspired by natural trees, an artificial trunk‐leaf device is built up to structurally mimic natural trees for autonomous bottom‐up flow and remote harvesting of water powered by sunlight.

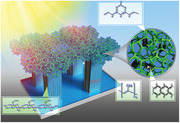

## 1 Introduction

Antigravity transport of water over a 100 m operates daily in natural trees powered by sunlight through a passive, wicking mechanism.^[^ ^1^ ^]^ This mechanism stems from the liquid surface tension and the difference in osmotic pressure in the tube‐like xylem. To optimize the rate and height of antigravity water transport, natural plants develop directional meters‐long microchannels built up from lignin, hemicellulose and cellulose, and the hierarchical network of leaf veins.^[^ ^2^ ^]^ Artificial structures mimicking natural trees show promising potential for continuous antigravity water delivery driven by solar energy.^[^ ^3^ ^]^ Although several groups have experimented on it with precise designs of gradient in surface energy and Laplace pressure, a continuous, long‐distance, and antigravity transport is not yet possible.^[^ ^4^ ^]^

**Figure 1:**
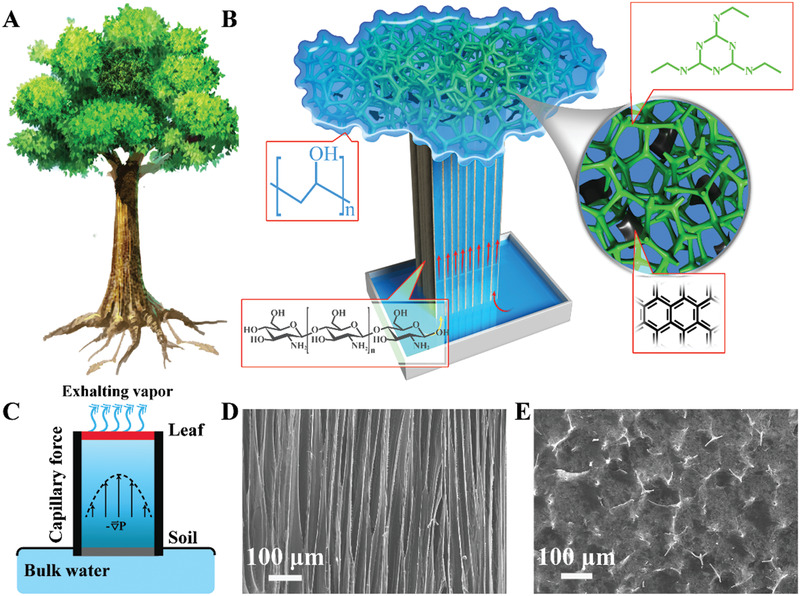
Design and characterization of the artificial trunk‐leaf system in trees. A) A schematic illustration of a tree, where the root absorbs water and sends it to the leaves via capillary force and simultaneously the leaves export water through transpiration and guttation. B) Schematic demonstration of continuous antigravity water transport in an artificial tree based on the directional microchannels in the artificial trunk with a photothermal steam generator on the top leaf. The water capillary rise is performed in acetylated chitosan channels, and a double network hydrogel consisting of poly(vinyl alcohol) (PVA), melamine‐formaldehyde (MF), and reduced graphene oxide (rGO) generates continuous water flows. C) Schematic diagram of a passive conduit for water to travel from the bottom to the air in an artificial tree under illumination of sunlight. Within the xylem, water rises driven by the capillary force into the leaf part, and then diffuses into the air by evaporation. The loss of water molecules by evaporation contributes to the built negative pressure (∇*P*) to pull liquid water up vertically. D) SEM image of the freeze‐cast acetylated chitosan matrix showing align channels. E) Cross‐section SEM image of the microstructures of the artificial leaves consisting of the MF foam, the PVA hydrogel and the incorporated rGO.

The capillary rise without using metabolic energy carries water from roots to leaves (**Figure** **1**A).^[^ ^5^ ^]^ According to the cohesion‐tension theory, the average value of the xylem pressure is −0.0096 ± 0.0007 MPa m^−1^ for trees of 110 m tall, which is identical to the hydrostatic gradient derived from gravity (−0.0098 MPa m^−1^).^[^ ^6^ ^]^ The absolute negative pressure driving water along the xylem originates from surface tension upon water adhesion to the channel walls and the continuous transpiration powered by sunlight.^[^ ^7^ ^]^ Strikingly, long microchannels and water evaporation are key to natural trees to maintain their internal water balance and continuous bottom‐up flow.

Herein we design and operate an artificial tree that enables continuous, long‐distance, and antigravity water transport powered solely by sunlight. Inspired by nature, we combined directional microchannels with a distal hierarchical leaf‐like hydrogel as a photothermal steam generator. To mimic the long‐distance tube‐like structures of the tree trunk, a chitosan‐based microchannel assembly was first fabricated by ice‐templating method followed by in situ acetylated into β‐chitin that is mechanically stable against water infiltration. The artificial leaf contains a double network of a polyvinyl alcohol/reduced graphene oxide (PVA)/rGO composite hydrogel and a melamine‐formaldehyde (MF) foam scaffold. Such a network promotes water suction and flow due to the engineered hierarchical water pathways at multiscale level, which in turn expedites water uptake and distribution inside the artificial leaf for enhanced photothermal conversion. Equally important, we demonstrate that the evaporative cooling and sufficient interfacial heat exchange can yield water steam collection in a high efficiency, finishing a complete water transport–evaporation–recollection cycle.

## 2 Results and Discussion

### 2.1 Synthesis and Characterization of the Artificial Tree

The basic operation principle of our artificial tree lies in the capillary rise of water via the designed microchannels, which are in direct connection to a hydrogel network for continuous water evaporation powered by solar irradiation (Figure 1B). Figure 1C is a simplified model of an artificial tree that supports the sunlight‐driven water transport. In the reservoir, water is drawn by capillary rise through microchannels into a leaf‐like structure to be photothermally transpired into air. The continuous loss of water by evaporation reduces the pressure of water inside the trunk; this built‐in negative pressure pulls up water continuously out of the reservoir to the leaf–air interface.^[^ ^8^ ^]^

The synthetic route to the artificial trunk and leaf is shown in Figure S1 of the Supporting Information. We employed a soft silicone tube as a trunk model to construct meters‐long microchannels. Microchannels were prepared by freeze‐casting an aqueous chitosan solution confined in the silicone tube (Figure S1, Supporting Information). Directional growth of ice crystals guides and assembles chitosan into aligned channels, which is easily controlled by monitoring the ice/liquid advancing rate.^[^ ^9^ ^]^ After freeze‐drying, the chitosan microchannels were in situ acetylated into β‐chitin by acetic anhydride in a methanol solution to build up mechanically and chemically more stable channels for water transport (Figure S2, Supporting Information).^[^ ^10^ ^]^ Under our reaction condition, a full acetylation ended up in 5 h. Infrared spectra of chitosan, the partially acetylated chitosan, and the as‐prepared β‐chitin show a polysaccharide‐dominated pattern (Figures S3 and S4, Supporting Information). Upon acetylation, a new single band at 1653 cm^−1^ emerges that is assigned to stretching the hydrogen bond between the C=O group and the adjacent N—H group.^[^ ^11^ ^]^ Additionally, the carbonyl stretching (*v*_c = o_, amide I) band splits at 1626 cm^−1^, and a new NH bending (δ_NH_, amide II) occurs at 1560 cm^−1^. Along the acetylation reaction, the surface hydrophobicity increases from 15.2° for pristine chitosan to 47.6° for fully acetylated sample. Figures S5 and S6 of the Supporting Information visualize the observed shrinkage occurring in the partially acetylated samples (reaction time from 1 to 4 h) when immersed in water. By contrast, the fully acetylated samples (reaction time of 5 and 6 h), i.e., the chitin one, has deformation of less than 5%. We also investigated the effect of bending on the tensile strength. The tensile strength remained nearly unchanged upon repeated bending for 15 cycles (Figure S6, Supporting Information).

SEM images of the resultant chitin trunk structure prove a unique directional tubular state (Figure 1D), which bear a remarkable resemblance of a natural tree (Figure S7, Supporting Information). Their tunable diameters depend on the concentration of aqueous chitosan solutions used in the ice‐templating method. Upon increasing the concentration of the chitosan solution from 0.5 to 1.0, 1.5, and finally 2 wt%, the average diameter of the channels produced by freeze‐drying shrinks from 160 to 80, 60 and 20 µm, respectively (Figure S8, Supporting Information). To be noted, though β‐chitin crystal is known stable in water, it features a large number of intrasheet hydrogen bonds,^[^ ^12^ ^]^ allowing water to quickly pass through without disturbing their microstructure. Finally, the as‐synthesized chitin matrix was treated with H_2_O_2_ to improve the surface hydrophilicity (Figure S9, Supporting Information). Thus, hydrophilic and mechanically stable chitin microchannels were assembled inside the artificial trunk.^[^ ^13^ ^]^ As observed in Movie S1 of the Supporting Information, the trunk swelled in water rapidly within seconds (Movie S1, Supporting Information).

A natural leaf has hierarchical interconnected veins for convenient water transfer and heat exchange along the pathways. As we demonstrated previously, the porous and superhydrophilic MF skeleton accelerates water allocation to the leaf–air interface to be solar‐steamed.^[^ ^14^ ^]^ A photothermal steam evaporator that mimics the leaf structure is thus prepared by incorporating a PVA hydrogel inside the MF foam (Figure 1E). The entire MF skeleton facilitates uniform water flow and is connected with the open channels in the PVA hydrogel, a structure in high similarity to that in the natural leaves. Such an interconnecting vein‐like design serves as water transport highways for efficient utilization of the invested light. Once water is carried via capillary rise through the artificial trunk to the PVA/rGO composite hydrogel leaf, the superhydrophilic porous MF foam enables a stable and uniform water supply to the leaf–air evaporation surface.^[^ ^15^ ^]^ The SEM images (Figure S10, Supporting Information) showed the morphology of the freeze‐dried hydrogel at different PVA loading content. The pore size inside the dried hydrogel decreased with increasing PVA loading. At a 10 wt% loading of PVA, pores of 20 ± 3 µm in diameter formed inside the MF skeleton. In a final step, the evenly distributed GO nanosheets were in situ reduced by hydrazine to rGO to further improve photothermal conversion. The additive rGO nanosheets simultaneously enhance light absorbance and toughness of the prepared PVA hydrogel (Figures S11 and S12, Supporting Information). Furthermore, the introduction of the MF foam not only provided the desired enhancement in toughness, but also maintained the shape and size of the leaf while being irradiated by light. The resultant leaf has enhanced mechanical properties and can withstand a much higher stress than that purely made up from PVA hydrogel (Figure S13, Supporting Information). In contrast to the pure hydrogel system, the high strength of the double network structures leads to the high mechanical stability of the synthetic leaf, avoiding being damaged by solar irradiation.

### 2.2 Antigravity Water Transport Performance of the Artificial Tree

**Figure 2:**
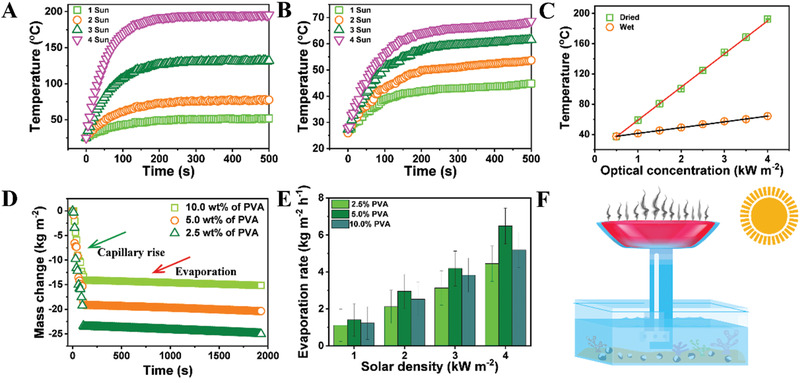
Performance of antigravity water transport. The surface temperature fluctuation of A) dried and B) wet hydrogel surface under sun irradiation of different intensity. Dried samples were obtained by freeze‐drying their wet counterpart. Wet samples were prepared by dialyzing the freshly fabricated PVA/MF hydrogel against pure water at room temperature, with the water changed twice a day. C) The temperature change of dried and wet artificial leaves under solar illumination of different intensity. Error bars represent the standard deviation of the experimental data measured at least three times, and they are smaller than the symbol size. D) Real‐time monitoring of water transport in an artificial tree with a height of 10 cm. E) Evaporation rate of water based on artificial leaves with various contents of PVA under different illumination intensity. F) Experimental setup for testing the performance of water transport. The artificial tree was held by a metal support. Mass loss of the water reservoir is monitored by an electronic balance.

To investigate the photothermal conversion performance under sunlight illumination, we used infrared camera (Fluke) to monitor temperature variation of the artificial leaf. **Figure** **2**A compares the surface temperature profiles of the wet and dried hydrogel under increasing intensity of light irradiation. In terms of the wet leaf, the temperature rose as the solar irradiance started and reached a steady state after 200 s, which is comparable to previous reports.^[^ ^16^ ^]^ By contrast, the temperature peaks within 10 s for the dried foam. This sharp increase of the temperature indicated that the integrated rGO is capable of quick photothermal conversion. For a quantitative analysis, the temperature of the leaf under various solar irradiation was monitored by the infrared camera. Under solar irradiation of 1 to 4 kW m^−2^ (i.e., 1 to 4 sun), the final steady surface temperatures up to 64.3 and 192.5 °C were recorded for the wet and dried samples, respectively (Figure 2B,C; Figures S14 and S15, Supporting Information). The ultrafast temperature rises, and the rather high final temperature well above 100 °C in the dried leaf proves the high efficiency of our materials design for the artificial leaf.

The continuous water transport in our artificial tree was quantified by measuring its weight loss with a setup shown in Figure 2F. Water was directionally sucked from the bottom reservoir to the top leaf, which was monitored by a digital camera in a time‐lapse mode. Images were taken as long as the bottom of the trunk was immersed into the water reservoir. A gradienter (LETA) was employed to determine the vertical direction of water transport. It is observed that the maximum height decreases logically as the average diameter of the channels increases, in agreement with the Lucas–Washburn equation.^[^ ^17^ ^]^ Compared with the rate of sunlight driven evaporation, water loss due to the capillary rise (green arrow in Figure 2D) is much faster (almost two orders of magnitude higher) than solar steam generation (red arrow in Figure 2D). Thus, one can construct an artificial tree that consists of a single trunk connected to a number of leaves, similar to a natural tree carrying many leaves on its trunk.

Typical plots of time‐dependent mass change under solar irradiation were recorded, and the evaporation rates were obtained by calculating the slope of the curves. The intrinsic evaporating capacities of the artificial leaves are 1.60, 2.95, 4.19, and 6.49 kg m^−2^ h^−1^ under solar illumination from 1 to 2, 3, and 4 kW m^−2^, respectively (Figure 2E). Apparently, the PVA hydrogel with a proper porous structure can dramatically increase the efficiency of steam generation. The lower evaporation velocity in the hydrogel with an average pore size of 250 µm may arise from the suppressed water distribution.^[^ ^18^ ^]^ In this context, water distribution is accelerated by the hierarchical water pathways constructing both the PVA hydrogel and MF scaffold. The optimized water evaporation velocity of 1.6 kg m^−2^ h^−1^ achieved in the artificial leaf is a hydrogel with an average pore size of 10 µm, which is made from a PVA solution of 5.0 wt%.

**Figure 3:**
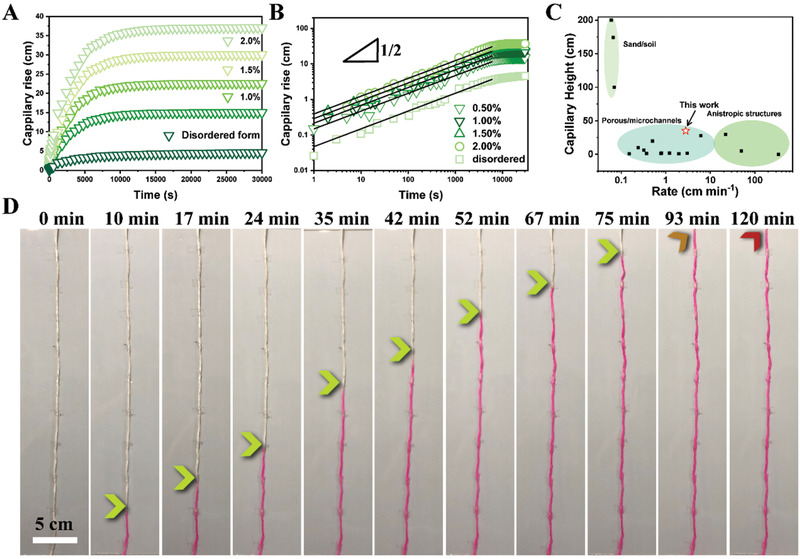
Water transport behavior in the artificial trunk. A) Real‐time monitoring of the antigravity water transport in tubes with microchannels of various diameters. B) The relationship between the rising height and time in a log–log plot, showing a 1/2 power scaling effect. C) Comparison of capillary rise and transport rate of water obtained by previous reported researches.^[^ ^18^ ^]^ D) Optical images of a water capillary rise inside an artificial trunk.

As observed by a digital camera, the height of water transport along the trunk (termed *H*_w_) depends on the characteristic diameter of its microchannels. By decreasing the diameter (i.e., by varying the concentration of the chitosan solution from 0.5 to 2.0 wt%), both *H*_w_ and the evaporation rate increase (**Figure** **3**A,B; Figure S17, Supporting Information). The transport velocities of water in various microchannels were analyzed statistically toward the structural features (Figures S16 and S17, Supporting Information). The water could be rapidly lifted up within a short period of several min by capillary force. Compared with previously investigated similar structures including soil, sand, porous polymers, and other anisotropic structures, this trunk‐leaf design realized both continuous antigravity water transport and remote clean water harvesting (Figure 3C). Owing to its unique trunk‐leaf design, this biomimetic system delivers a high efficiency to convert solar energy to potential energy. It is a great challenge to achieve a high transportation and high‐efficiency steam generation in a single system, which needs to balance the rate of water transportation and steam generation, as well as water distribution inside the leaf part.^[^ ^19^ ^]^ In this work, the highly ordered microchannels, customer‐designed trunk numbers and double‐network hydrogels enables the artificial tree to obtain excellent ability of continuously lifting water by solar irradiation. For better visibility, the water in this test was dyed with Rhodamine B. The longest transport height of 37 cm was achieved in a first quick transport step of ≈2 h, followed by a slow transport step of ≈6 h. In most tests, the forefront water/air interface advanced with negligible disturbance from air bubbles trapped, as were detected visually (Figure 3D). The aligned microchannels, wrinkled inner walls and abundant surface hydroxide groups due to H_2_O_2_ treatment all contributed to the fast and long‐distance water transport. In control experiments, the highest water capillary rise in a porous chitosan aerogel and a porous acetylated β‐chitin aerogel without directional microchannels were only 11.5 and 9.8 cm, respectively.

### 2.3 Principle Analysis

Our further effort was devoted to a comprehensive understanding of the relationship between the rising height of water inside the artificial trunk and the evaporation rate from a theoretical point of view. The driving force originates from the capillary force, 2σcosθ/*r*, where σ denotes the surface tension of water, θ is the contact angle, and *r* is the characteristic radius of the pores in the microchannels of the tube. During the transport, the drag force results from the viscosity of water in motion, i.e., *εμHU*/*K*, where ε denotes the material porosity, *K* is the permeability of the material, μ is the viscosity of water, *H* is the rising height, and *U* is the corresponding vertical velocity defined by *U* = d*H*/d*t*. Since the rising height of the liquid is much larger than the capillary length,^[^ ^20^ ^]^ we have to consider the pressure *ρgH* resulting from the gravity, where ρ and *g* are the density of water and the acceleration of gravity, respectively. These analyses arrive a force balance along the tube in terms of the Lucas–Washburn equation^[^ ^17b^ ^]^

(1) $$\begin{array}{c} \frac{2\sigma \cos\theta }{r}=\frac{\varepsilon \mu }{K}\;H\frac{\text{d}H}{\text{d}t}+\rho gH \end{array}$$

By using *a* = (*K*/*εμ*)(2σcosθ/*r*) and *b* = *ρg*(*K*/*εμ*), we rewrite Equation (1) into a simpler formula *a* = *H*(d*H*/d*t*) + *bH*. Taking into consideration of the boundary condition *H*|*_t_*_= 0_ = 0, we get the analytical solution of the rising height *H*(*t*) as a function of time *t*, i.e., *t* = − (*H*/*b*) − (*a*/*b* ^2^)ln(1 − *bH*/*a*).

As shown in Figure 3B, our theory follows the experimental data very well which shows a 1/2 power scaling. Next, as shown in Figure 3C, we estimate the mass change during evaporation regarding the tube with different diameters. During evaporation, the heat transfer *q*_T_ through the water–air interface can be expressed as

(2) $$\begin{array}{c} q_{\text{T}}=\frac{\text{d}Q_{\text{T}}}{\text{d}t}\;=\;L\;\left(\frac{\text{d}m}{\text{d}t}\right)=\;\Delta Th_{i}A \end{array}$$

where *Q*_T_ and *m* denote the energy released and the mass of water during the phase change (from liquid to vapor) process, *L* is the latent heat of vaporization of water, Δ*T* is the temperature difference between water and the vapor phase, *h*_i_ is the interfacial heat transfer coefficient, and *A* is the cross‐sectional area of the tube. Then we can estimate the mass change during the evaporation *m* = (Δ*Th*_i_ *A*/*L*)*t*, which is a linear function of time *t*. This linear dependence is confirmed by the experimental data shown in Figure 3C (more details are given in Notes S1 and S2 and Figure S18, Supporting Information). These results suggest that by decreasing the characteristic pore size of the microchannel (in other words, by increasing the concentration of chitosan solution for the freeze‐casting process, see Figure S2, Supporting Information) it is capable of increasing the rising height. Meanwhile, in order to enlarge the mass change, tubes with a large cross‐sectional area should be preferably used.

**Figure 4:**
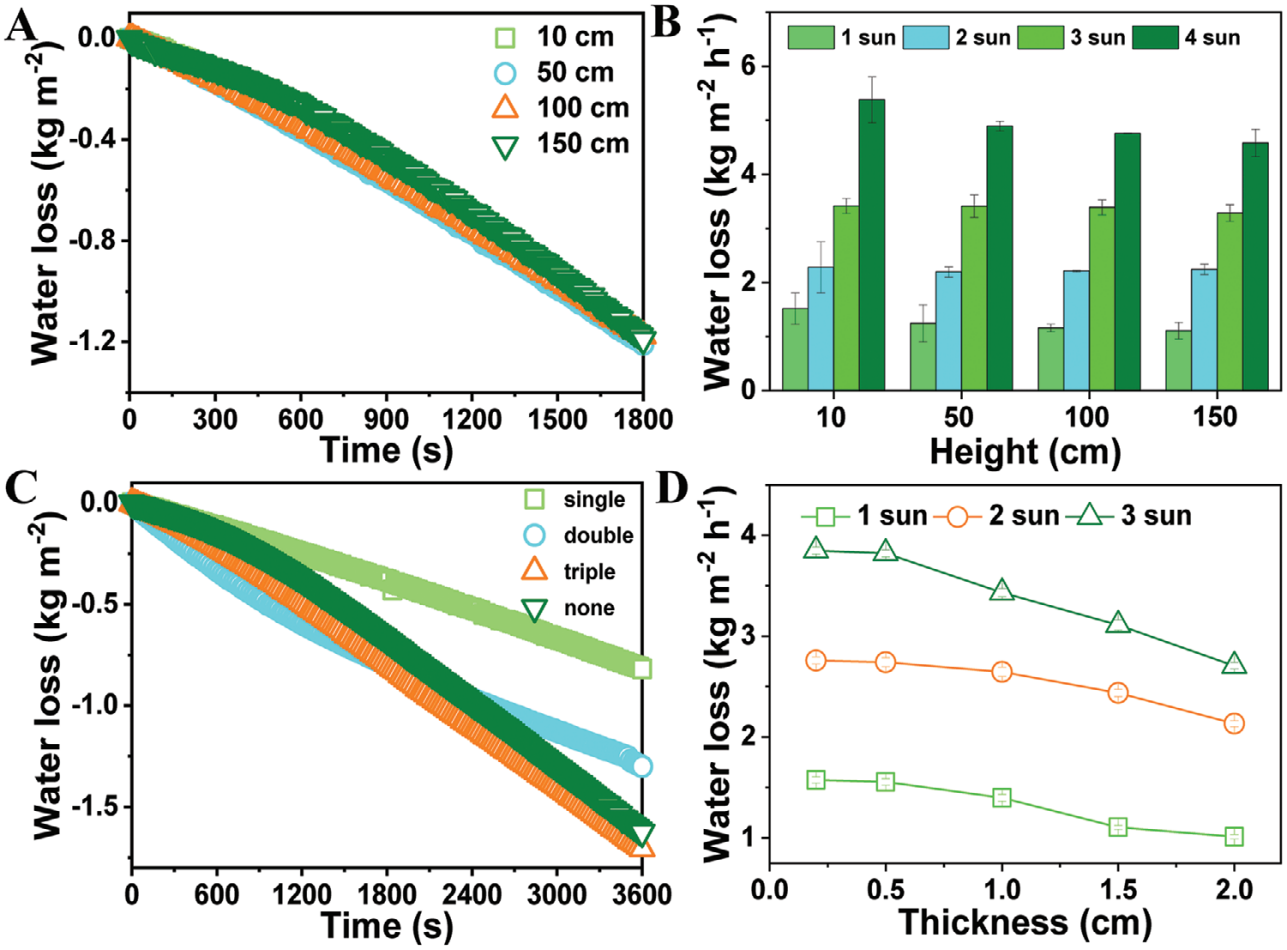
Performance of the artificial tree in antigravity water delivery. A) Real‐time monitoring of mass change of water over time when transported in an artificial tree with various heights under 1 sun illumination. B) Mass loss of water using artificial trees of various heights at different light intensities. C) The evaporation mass loss of water using artificial leaves with different number of trunks. D) The effect of leaf thickness on the efficiency of water evaporation at different light intensities.

The height, number of trunks, and thickness of leaves were further investigated by monitoring the rate of water transport and solar steam generation. As water capillary rises and directly contacts with the leaf part, the evaporation rate does not depend on the height of the artificial tree (**Figure** **4**A). As shown in Figure 2D, the velocity of water transport via capillary rise is much higher than that via evaporation, making the water evaporation from the leaf the rate determine step for antigravity water delivery. The mass loss under enhanced solar irradiation also shows less‐to‐no height dependence, but on the interfacial area between the trunk and the leaf (Figure 4B,C). Those branches were connected by the MF foam to the leaf part (Figure S19, Supporting Information). The fluidic stream in these microchannels was affected by various parameters including the physicochemical properties of the fluidic phase (e.g., viscosity, surface energy, and interfacial tension), diameter of the microchannels and wettability of the wall surface. At a constant trunk diameter of 6.0 mm, as the number of branches increases from 1 to 3, the velocity of mass change increased from 0.8 to 1.6 kg m^−2^ h^−1^ (Figure 4C). It is clearly shown that the velocity of mass change of a leaf without trunk is the same as the leaf with three branches. Further experiments showed that if branches were attached to a leaf in diameter of 20 cm, the velocity is ≈1.6 kg m^−2^ h^−1^ due to the mismatch of the velocity between the water diffusion inside the hydrogel and water transport by the capillary rise. The artificial leaves of 0.5 cm in thickness appear to be the optimum for the full absorption of input solar energy as well as efficient water distribution to the top surface of the hydrogel. As the thickness decreased down to below 0.25 cm, the leaf is semitransparent and light partially passes it through, so the maximum temperature is lower than that in thicker samples (Figure 4D).

To investigate the effectiveness of the ice‐templated microchannels on water transport, we carried out in situ observation of a water diffusion process along directional microchannels. A water droplet (≈1 mL in volume) deposited on the microchannels displays a dominant propagation of the liquid along channels, giving a fast wetting process from one channel to the other through the interstitial pores in the wall. Overall continuous and fast water diffusion is observed to cover the screen within 60 s along the microchannels (Movie S2, Supporting Information). In striking contrast, water transport in disordered chitin porous foams occurs simply through filling the cavity, resulting in a low propagation rate of only 1.25 × 10^−3^ cm s^−1^ (Movie S3, Supporting Information). Wettability measurement shows that the water contact angle (WCA) of acetylated β‐chitin is 58°. Chemical treatment in H_2_O_2_ solution can substantially reduce the WCA down to 0°, though only the surface of the chitins is modified with hydroxyl groups owing to the insolubility of acetylated chitin in water (Figure S9, Supporting Information). This superhydrophilic surface speeds up the water‐spreading rate, which is comparable with that of the peristome surface of *Nepenthes alata*.^[^ ^21^ ^]^

### 2.4 Remote Water Harvesting

**Figure 5:**
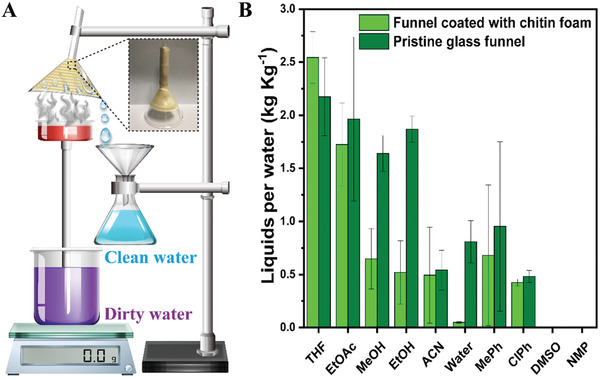
Collection of water steam produced by the artificial tree. A) Illustration of evaporative and cooling experimental setup. B) The consumption of liquids for the condensation of water steam.

To complete the cycle of long‐distance water transport, water evaporated from the artificial leaf needs to be recollected. It is known that evaporation cooling allows for thermal energy removal by liquid–gas phase change without extra fuel and electricity.^[^ ^21^ ^]^ In our design, an evaporative cooling system using organic liquids to condense steam was built up to recollect water vapor, as shown in **Figure** **5**A. The glass funnel was coated with a porous acetylated chitosan on its outside surface to improve its contact with organic solvent and was deployed above the leaf to collect water vapor. Evaporation of organic liquids on the outside surface of the funnel absorbed heat to cool down the funnel and thus precipitate water steam on its inside surface simultaneously. The evaporation rate of various liquids was calculated at ambient condition under 1 sun irradiation, as shown in Figure 5B and Figure S20 (Supporting Information). The equilibrium temperature of the funnel due to the liquid–gas phase transition of various liquids could be regulated in a wide range from 32.2 to 5.5 °C (Table S1, Supporting Information). As expected, evaporation rates of many organic liquids are much higher than that of pure water, through which the water steam could condensate at a lower temperature (Figure S21, Supporting Information). Heat released by the water steam condensation was harnessed to evaporate the organic liquids and regenerated the dried porous foam.

The porous chitin foam significantly improves the heat‐exchange between the organic liquid side (outside surface of the funnel) and the condensation side (inner side surface) by spreading the liquid drops to the entire outside surface.^[^ ^23^ ^]^ The water steam condenses on the inside surface of funnel, allowing for immediate collection of liquid water. Compared to the conventional solar steam generation system, our design comes up with an efficient way to recollect water vapor. Taking water and ethanol as an example, the collection efficiency is ≈75%. For collection water of 1 kg, the needed amounts of ethanol and water are 0.5 and 0.2 kg, respectively (Figure 5B; Figure S22, Supporting Information).

Generally, our tree‐leaf system overcomes diffusion limitation and lowers down transport resistance which allows long‐distance antigravity water transport powered solely by sunlight. Although previous reports, such as the manipulation of liquid in magnetic microactuators,^[^ ^4a^ ^]^ topological fluid diode for long‐distance transport of liquid,^[^ ^24^ ^]^ and photoresponsive manipulation of liquid slugs in crystal polymer microactuators,^[^ ^25^ ^]^ reported water‐transport capability, their transport distance usually is limited to a low value and/or a horizontal direction. This meters‐long artificial trunk fabricated using the ice‐template method has large‐scale structure uniformity, whereas it uses chitosan only 0.2 g m^−1^ to establish the transfer channel. Our device represents a concrete reliable step forward for solar powered controllable long‐distance water delivery; it could provide a potential solution to the currently imbalanced distribution of water resource.

## 3 Conclusion

We demonstrated antigravity water transport up to 184 cm powered by sunlight irradiation experimentally and theoretically. The artificial tree not only enables low cost of large‐scale uniform device, but also displays high efficiency of antigravity water transport (1.6 kg m^−2^ h^−1^ illuminated under 1 sun) and realizes 100% of steam recollection. The relevant parameters, such as diameter of the microchannels, height of the truck, and constitutional contents of leaves have been systematically investigated to optimize the overall performance. The corresponding results fit very well with our theoretical models, which suggests that decreasing the characteristic diameter of the microchannels could increase the rising height. This simple setup model enables a long‐range antigravity water transport and an efficient water recollection. These features imply that the sunlight powered artificial trees could open up a new horizon in continuous, long‐distance and antigravity liquid transport.

## 4 Experimental Section

##### Synthetic Trunk Fabrication

The synthetic procedure of the artificial trunk is reported previously with slight modification.^[^ ^10^ ^]^ Briefly, 0.5, 1.0, 1.5 or 2.0 g of chitosan (degree of deacetylation = 90%; from Shanghai Macklin Biochemical Co., Ltd) was dispersed in 96 mL of deionized water (DIW; 18.2 MΩ; Milipore). Simultaneously, 2 mL of glacial acetic acid was added dropwise into the dispersion with continuous manual stirring to form an optically clear solution. Air and bubbles were removed via by centrifuge at a speed of 3000 rpm for 5 min. In the meantime, the silicone tube that is 6 mm in inner diameter and meters‐long was slotted every 10 cm. The pores were sealed with paraffin. The as‐prepared chitosan solution was poured into this silicone tube. The filled silicone tube was precooled to 4 °C before fully freezing via liquid nitrogen. Precise control of the freezing direction along the long axis of the tube guarantees chitosan to form parallel channels in the silicone tube. By removing the paraffin to open the holes on the silicone tube, the sample was freeze‐dried. Then the dried chitosan in the tube was in situ transformed to β‐chitin by acetylation using a mixed solution of methanol and acetic anhydride (volume ratio = 10:1) at 50 °C for 4 h. Afterward, the chitin matrix was placed in a boiling water containing 1 wt% of H_2_O_2_ for 2 h to obtain a superhydrophilic surface. The same procedure except for directional freezing was conducted to prepare a porous β‐chitin matrix.

##### Preparation of MF Foam

The foam was employed as the skeleton of artificial leaves and in conjunction between leaves and trunks to assure leakproofness and fast water transportation. The MF foam was prepared via a traditional alkaline‐acid two‐step reaction. First, 20.0 g of melamine and 38.2 g of formalin (37%) were mixed in a three‐neck flask under vigorous mechanical agitation (200 rpm). The mixture was heated to 60 °C, allowing the completeness of dissolution. Subsequently, the system was adjusted to pH 8.5 by NaOH (1.0 m). Then the solution was heated to 80 °C for 2 h to synthesize the MF resin. For preparation of the foam, a mixture was formed by adding 26‐(4‐octylphenoxy)‐3,6,9,12,15,18,21,24‐octaoxahexacosan‐1‐ol (OP‐10, 1.25 g) and *n*‐pentane (1.0 g) into the above mixture. The mixture was cooled below 10 °C. Then formic acid (curing catalyst, 1.0 g) was added and stirred quickly for 60 s. Finally, the obtained white viscous foam latex was quickly transferred into an oven preheated to 120 °C for 2 h.

##### Artificial Leaf Fabrication

The procedure follows the previous work with slight change.^[^ ^14a^ ^]^ 10 g of PVA was dissolved in 100 mL of deionized water at 90 °C under vigorous mechanical stirring. Then 1.25 mL of glutaraldehyde (50 wt% in deionized water), HCl (0.5 mL, 1.2 m) and 5 mL of graphene oxide aqueous dispersion (5 mg mL^−1^) were added and mixed by mechanical stirring and sonication. The resulting dispersion was degassed and poured into the MF foam. The gelation was carried out at room temperature for 2 h. The resultant hydrogel was transferred to a sealed beaker, into which several droplets of hydrazine hydrate were added. The chemical reduction of graphene oxide was then performed at 90 °C for 2 h. Finally, the obtained hydrogel was immersed in water overnight for several times to remove unreacted reagents.

##### Observation of Water Transport

The experimental setup was schematically illustrated in Figure 2F. A silicone tube filled with a directional β‐chitin (acetylated chitosan) matrix or a disordered porous β‐chitin matrix was vertically connected by its lower extreme to a water reservoir which contained 0.01% of Rhodamine B for easy visualization. At the beginning of the experiment, the tube was dried, and antigravity water transport was initiated by the effect of capillary force. The height of the front was determined by a digital camera as a function of time. In order to eliminate fluid loss by evaporation during the capillary rise, a humidifier was connected to the top of the tube, allowing water saturated.

##### Water Harvesting Measurement

The remote water harvesting by the trunk‐leaf system was real‐time monitored via an electronic microbalance with an accuracy of 0.0001 g under irradiation of various power densities was measured using a simulated solar illumination. The whole artificial tree is held by an iron support to monitor the mass change on the balance. Solar steam generation was recorded by immersing the trunk in deionized water and connecting to the leaf via a printed connector. The remote water harvesting in the height of 184 cm (the same for 50, 100 and 150 cm) was performed by filling the trunk structure with water before connecting to the leaf part. All samples were placed inside a beaker allowing water (dyed with a small quantity of Rhodamine B) touched the bottom of the peristome of the resin tube containing β‐chitin microchannels. The front water/air interface was recorded by videos. Leaf structures with various thickness and diameter were put on the top of the trunk.

##### Calculation of Energy Efficiency

The efficiency for solar steam generation was calculated by the following equation

(3) $$\begin{array}{c} \eta \;=\;\frac{\dot{m}h_{\text{V}}}{C_{\text{opt}}P_{0}} \end{array}$$

where $\dot{\text{m}}$ represents the mass change, *h*_V_ refers to the vaporization enthalpy of water under standard atmospheric pressure. *C*_opt_ stands for the optical concentration (1–4 suns used to heat the leaf part), *P*_0_ is the power of 1 sun irradiation (1 kW m^−2^).

## Conflict of Interest

The authors declare no conflict of interest.

## Supporting information

Supporting Information

Supplemental Movie 1

Supplemental Movie 2

Supplemental Movie 3
