# Supplementary material for: Biomimetic Antigravity Water Transport and Remote Harvesting Powered by Sunlight
Source: Glob Chall. 2020 Sep 6;4(11):2000043. doi: 10.1002/gch2.202000043 (PMC7607244; doi:10.1002/gch2.202000043)
Supplement: Supplementary file 1 — Supporting Information [file GCH2-4-2000043-s001.pdf]

© 2020 Wiley-VCH GmbH

Global Challenges Open Access

Supporting Information

for *Global Challenges,* DOI: 10.1002/gch2.202000043

Biomimetic Antigravity Water Transport and Remote Harvesting Powered by Sunlight

Hongya Geng, Cunjing Lv, Mingmao Wu, Hongyun Ma, Huhu Cheng, Chun Li, Jiayin Yuan,* and Liangti Qu**

Supporting Information

Bomimeticanti-gravity water transport and remote harvesting powered by sunlight

HongyaGeng, CunjingLv, Mingmao Wu, Hongyun Ma, Huhu Cheng, Chun Li,*Jiayin Yuan,* and Liangti Qu*

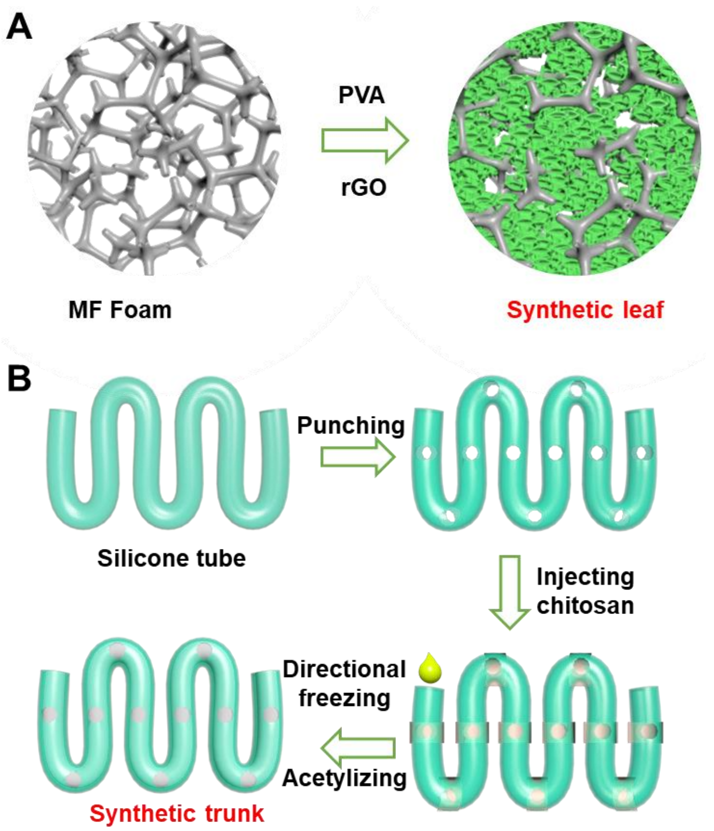

Figure S1. Synthetic approach to an artificial tree. (A) Schematic illustration of the fabrication process of the meters-long channel for water capillary rise. A silicone tube was chosen for the development of a self-stand and durable transport canal. The flexible property of the silicone tube is necessary for establishment of a meters-long structure. Ideally speaking, the length could be as long as possible.Before use, the silicone tube was punched with holes every ten centimeters, which were sealed off by paraffin. Subsequently, the tube was filled with an aqueous chitosan solution of defined concentration before fully freezing. For practical reasons, the tube was folded into a “M” shape to spatially fit the size of our freezing dryer. After freezing, the punched holes on the silicon tube were re-opened to accelerate the freeze-drying and to avoid melting down of the frozen chitosan solution. (B) Schematic illustration of the leaf part. PVA solution containing dispersed GO was poured into the MF foam. After gelation and chemical reduction, a black leaf structure was obtained.

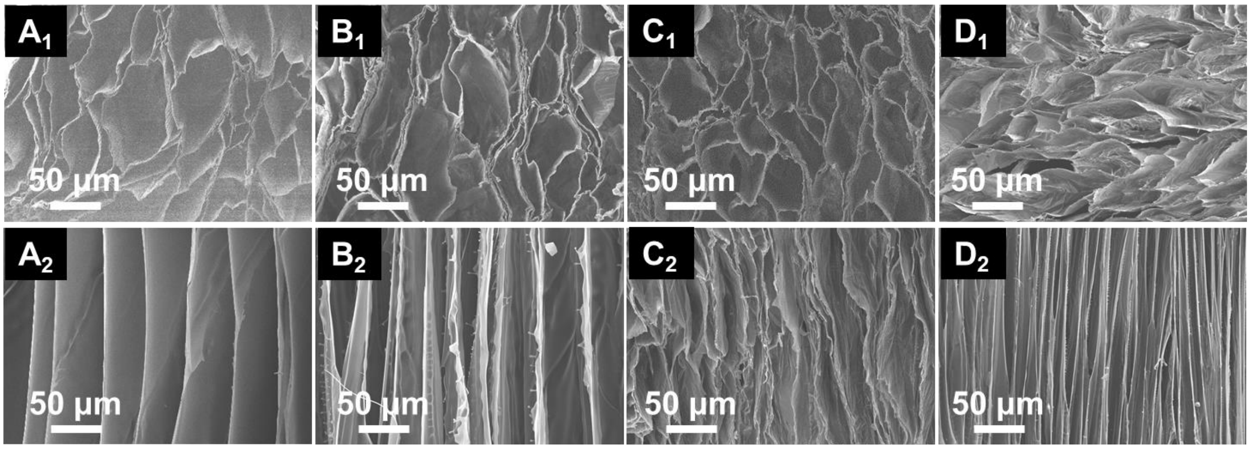

Figure S2. SEM images showing structural feature of the artificial trunk with align vertical channels. (A) to (D) Ice-templating laminated acetylated β-chitin matrix. The tubular spacing is regulated by the concentration of chitosan solution (shown in Figure S8). A1-D1 images are the top-view and A2-D2 are vertical view normal to the channels.

The stretchable silicon tube extends the ice template method to fabricate large scale trunk structures. The consumption of chitosan is estimated to be only 0.2 g m^{-1}. The uniformity of the meters-long synthetic trunk was examined and proven by measuring the diameter of both trunks and microchannels of the resulted tubes at several chosen sites. At a large-size scale, the diameter variation over the meter-long range was negligible, *i.e.* less than 5%. The ice-templating method using the chitosan solution provides a powerful technique to establish meters-long microchannels for long-distance water transport by tailoring the nucleation and growth of ice crystals. This filled tube also exhibited similar flexibility and durability to the pristine one. Compared with that of a shorter trunk, the continuous β-chitin structure engineered by ice-templating method resulted in a satisfactory structural uniformity along the silicon tube.

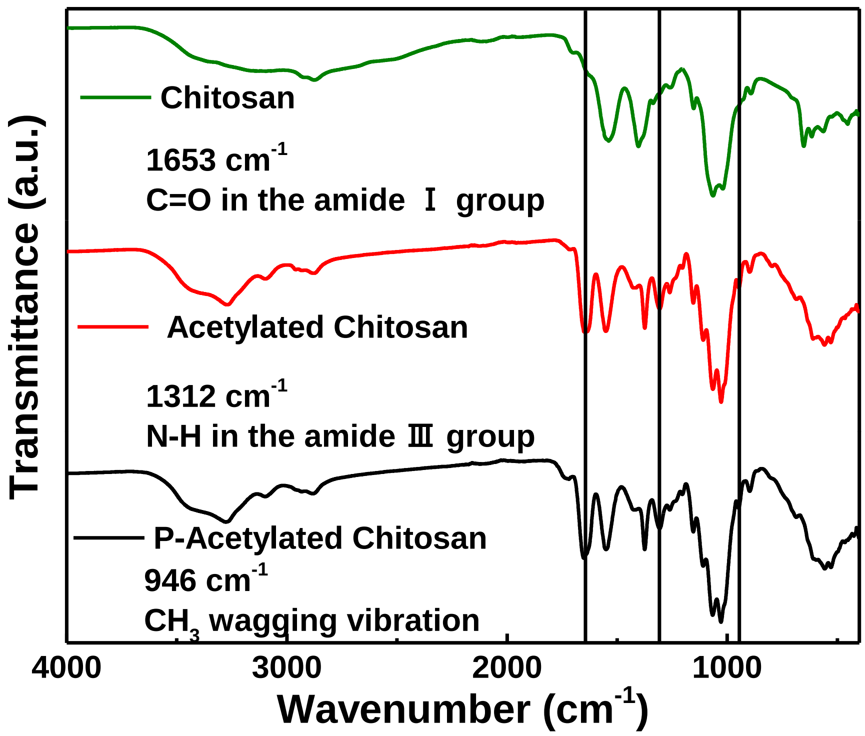

Figure S3. Infrared spectral of chitosan, the fully acetylated chitosan (chitin) after 5 h reaction time, and a commercially available partially acetylated chitosan sample (P-acetylated chitosan). The vertical lines highlight the bands of significant change.

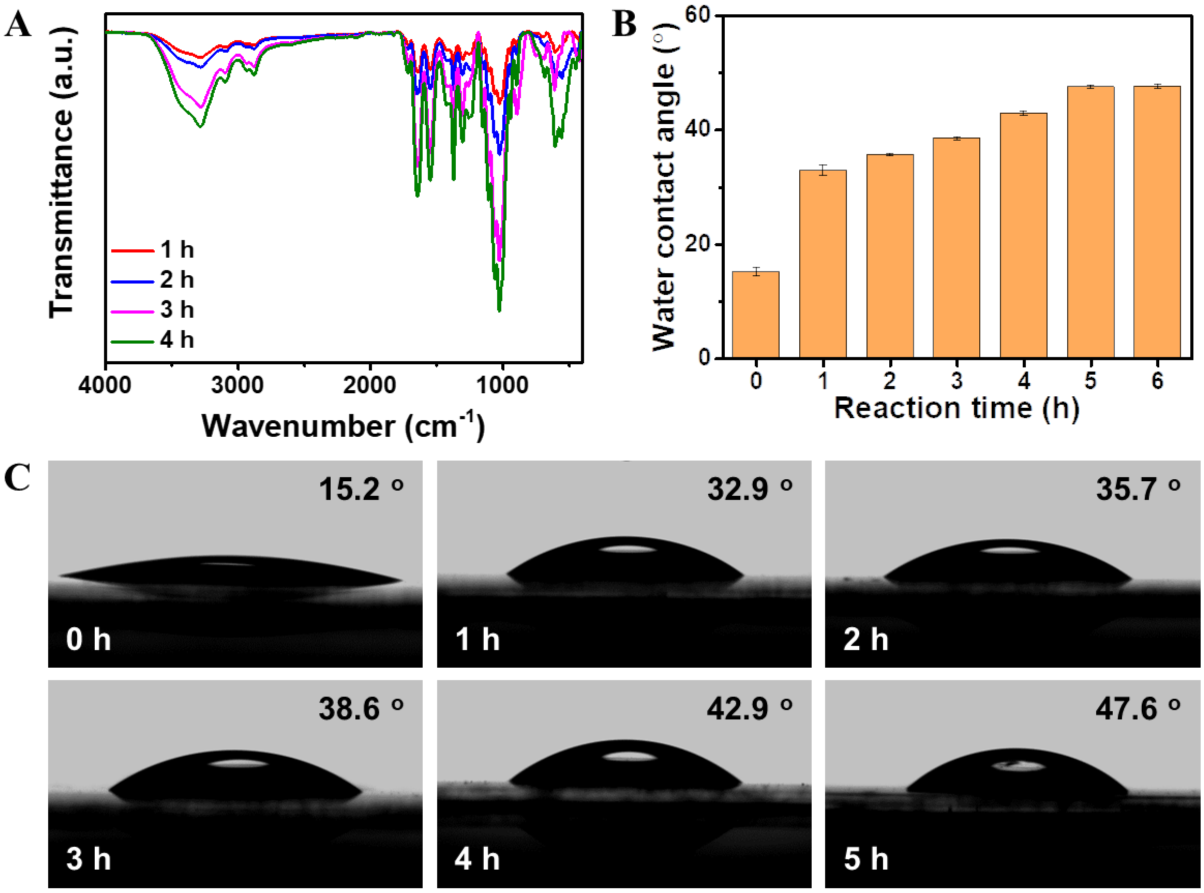

Figure S4. Acetylation reaction of chitosan into β-chitin along the reaction time. (A) Infrared transmittance spectra of the acetylated samples along the reaction time. (B) The plot of water contact angle *vs.* the reaction time in the acetylation reaction. The water contact angle was measured on the acetylated samples obtained along the acetylation reaction.

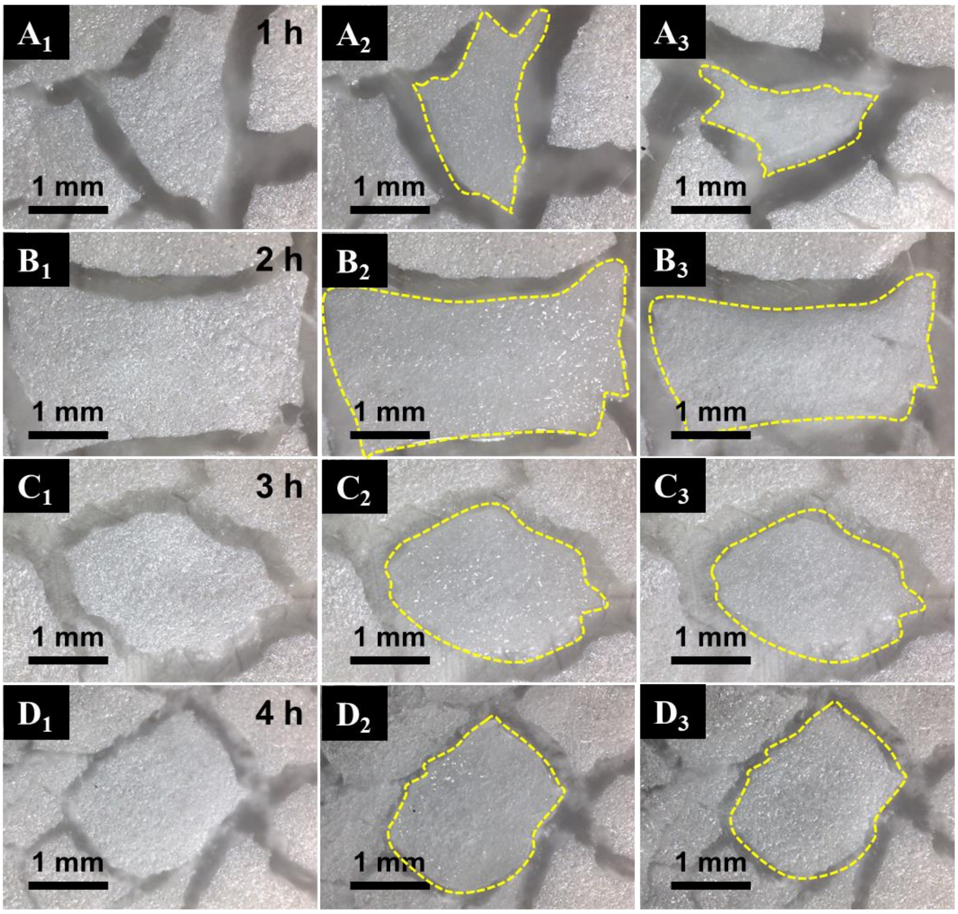

Figure S5. Shape variation of partially acetylated chitosan matrix after various reaction time (5h for fully acetylated sample). Digital images of partially acetylated chitosan matrix after various reaction time (A: 1, B: 2, C: 3 and D: 4 h). The photos were taken by immersing partially acetylated chitosan into water.

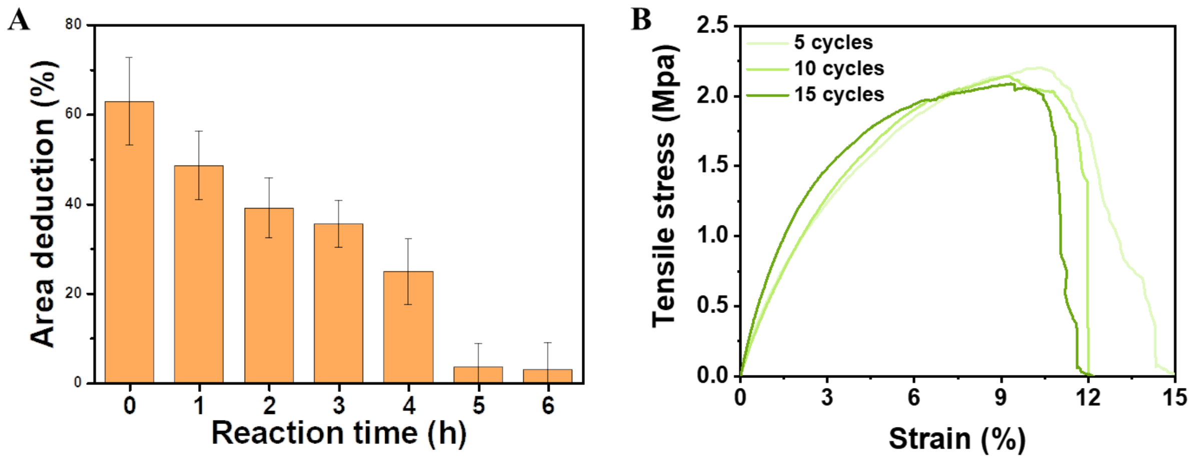

Figure S6. Structural stability of the synthetic trunk. A, Area deduction of partially (1-4 h) and fully (5 and 6h) acetylated chitosan after different reaction time. The area deduction is measured by immersing chitosan and partially acetylated chitosan samples in water, and the area deduction was monitored and determined. Reaction time t=0 represents the pure chitosan sample. Reaction time at 5 and 6 produced samples with less than 5% area deduction, indicating the reaction proceeded to the end with full acetylation. B, Mechanical stability test of repeated bending at 180o for 5 cycles, 10 cycles and 15 cycles, respectively.

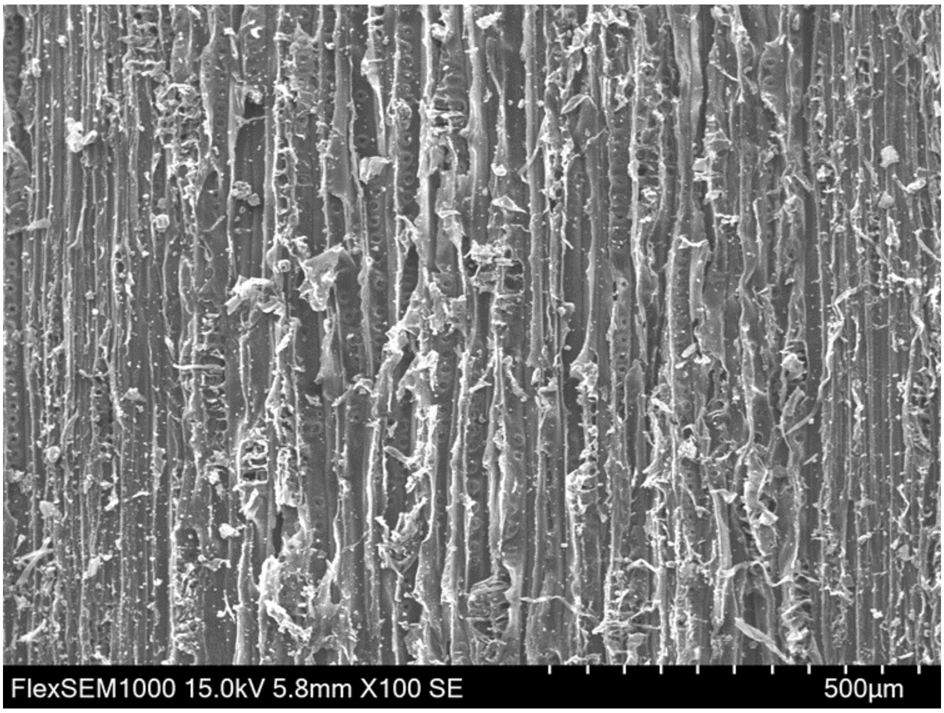

Figure S7. SEM image of a wood slab through vertical cutting. Aligned microchannels are visible.

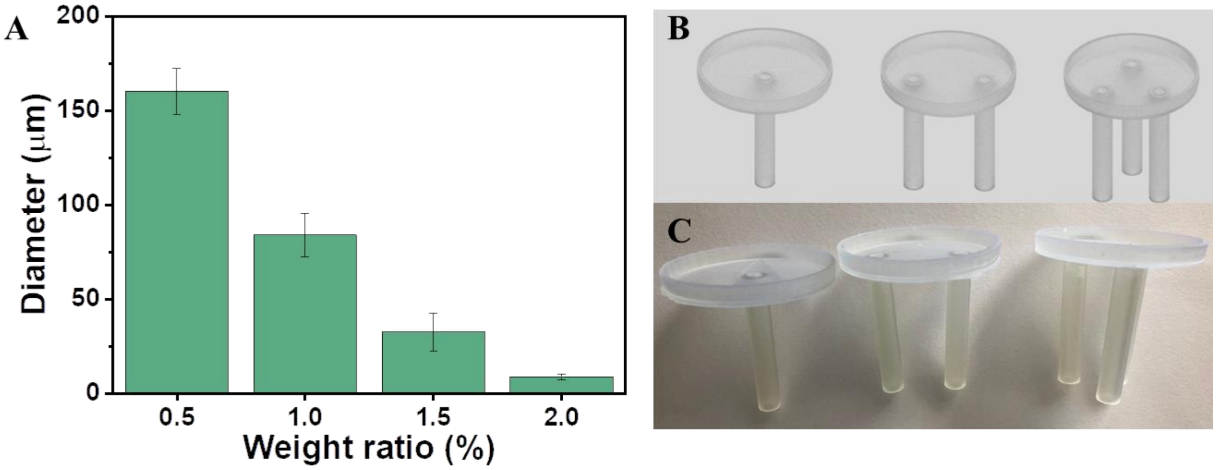

Figure S8. Dependence of the diameter of the microchannels in the synthetic trunk on the concentration of the chitosan solution. Diameters of the microchannelsare calculated from 100 microchannels. The diameter is apparently regulated by the concentration of chitosan solution from 0.5 wt% to 2.0wt%. A, Scheme for the connector of trunk and leaf. B, Digital photograph of as-prepared connector.

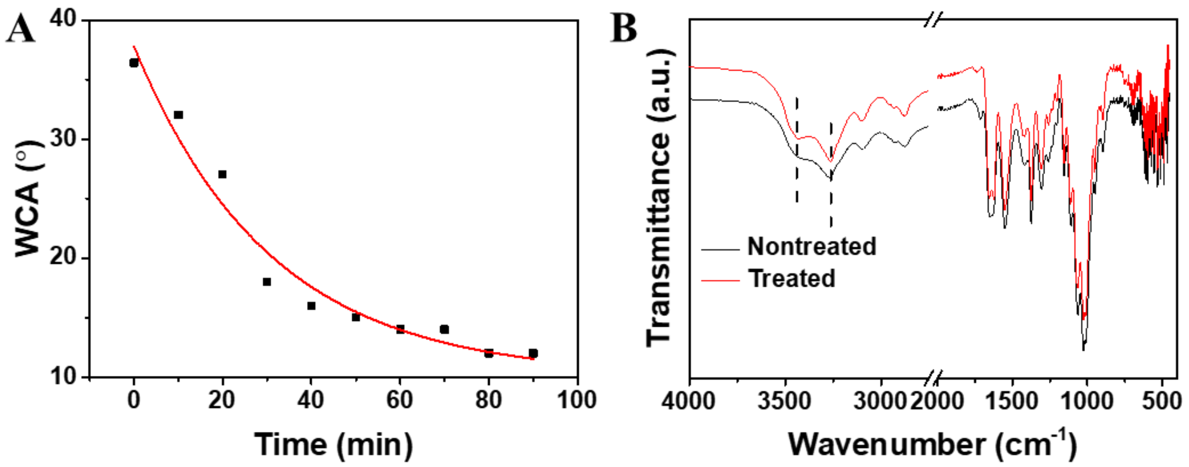

Figure S9. The regulation of surface wettability of the fully acetylated chitosan by H_2O_2 treatment. (A) Plot of water contact angle as a function of the H_2O_2 treatmenttime. The wettability of the acetylated chitosan was regulated by H_2O_2treatment for various time to get a high hydrophilicity. (B) Infrared transmittance spectra of theacetylated chitosan before and after the H_2O_2 treatment. This treatment enhances the OH band intensity in the range of 3250-2500 cm^{-1}, which improves the surface wettability.

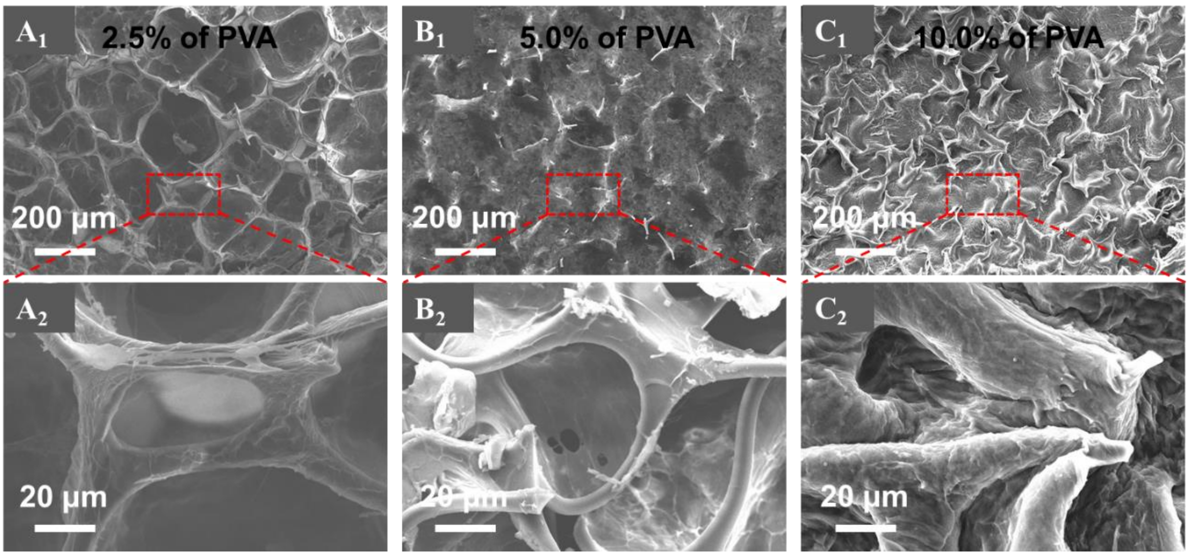

Figure S10. Morphologies of the PVA-MF foam-rGOcomposite leaf with various PVA contents. (A_1) Cross-sectional SEM image at 2.5wt% of PVA and the corresponding magnified image (A_2). (B_1) Cross-sectional SEM image at 5.0wt% of PVA and the corresponding magnified image (B_2). (C_1) Cross-sectional SEM image at 10.0wt% of PVA and the corresponding magnified image (C_2). The MF skeleton connects porous structuresforfast water distribution inside the PVA-rGOhydrogel.

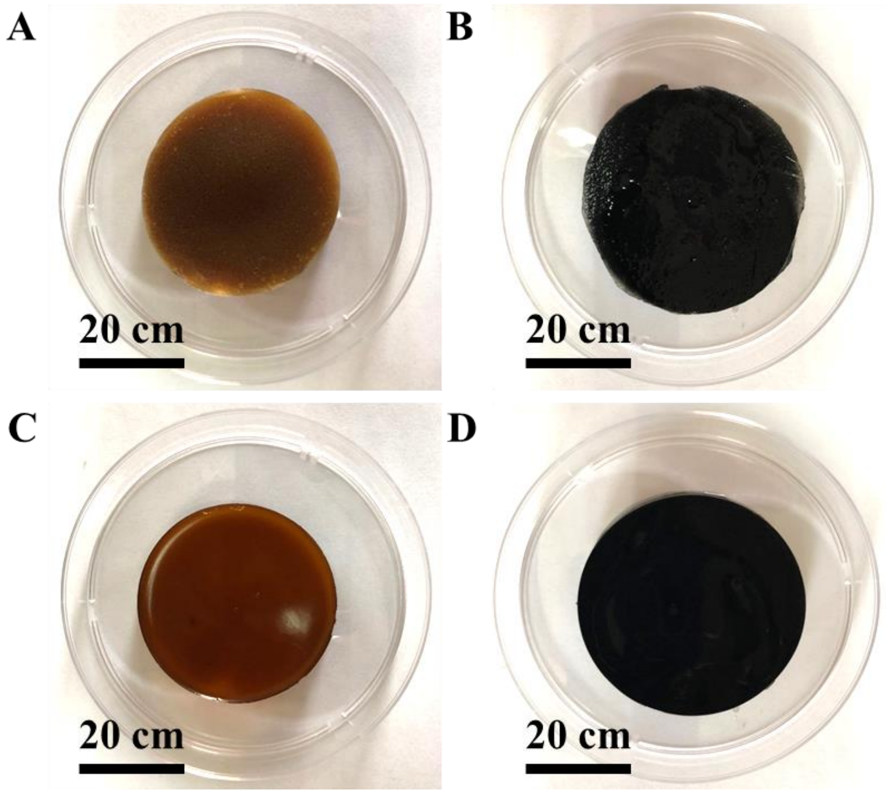

Figure S11. Digital images of (A) PVA-MF foam-GO hydrogel; (B) PVA-MF foam-rGO hydrogel; (C) PVA-GO hydrogel; (D) PVA-rGO hydrogel. It is visible that the rGO leaves (B & D)are darker than the GO ones (A &C). The MF form is needed for a btter mechanical stability against the induced stress during solarthermal conversion.

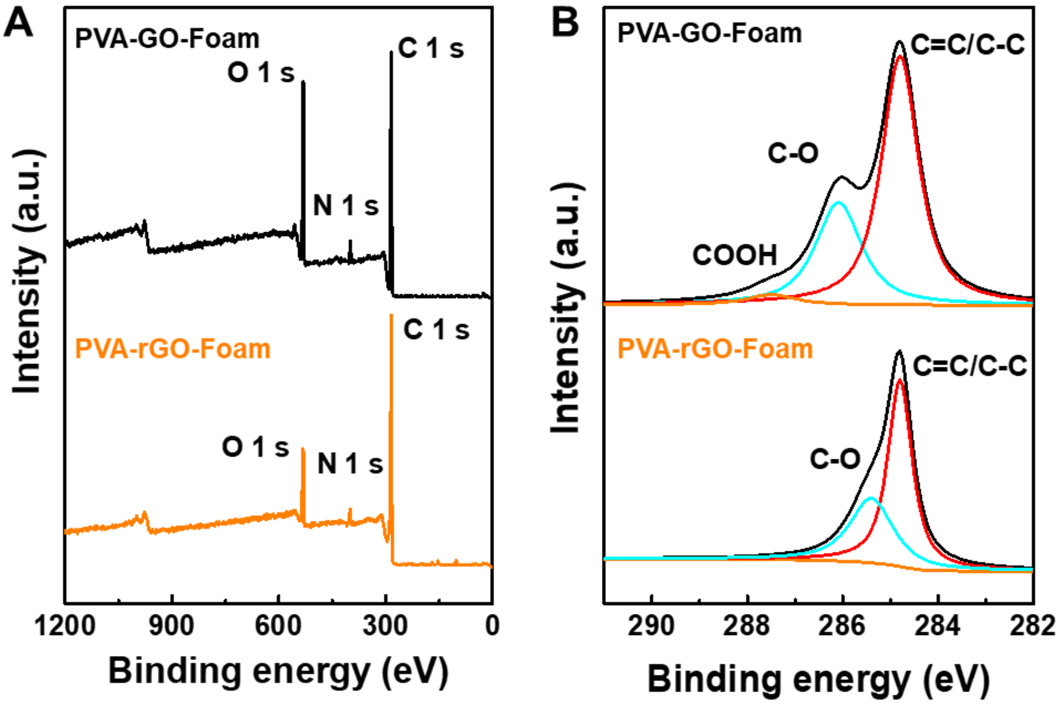

Figure S12. Characterization of the chemical structures of the leaves before and after chemical reduction of GO into rGO by hydrazine: (A) XPS spectra of the samples. (B) C 1s spectra of the samples.

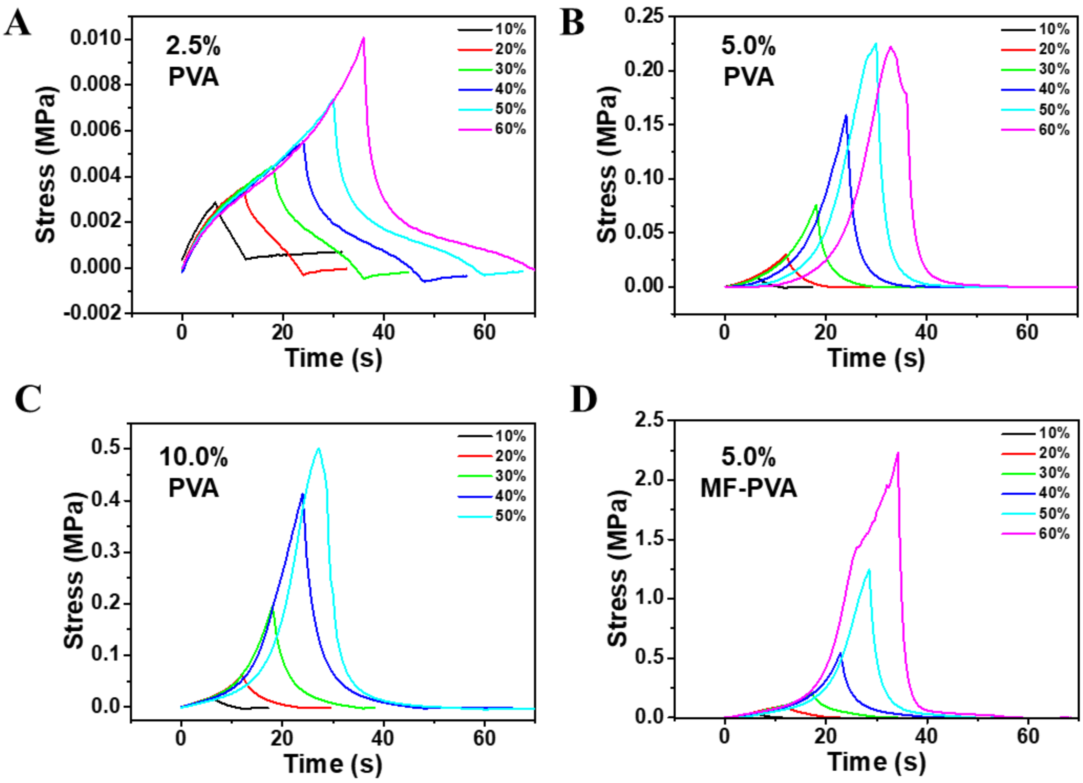

Figure S13. Mechanical compression tests of the PVA hydrogels with different PVA contents (2.5%, 5% and 10% in A, B and C, respectively) and the PVA hydrogel (at 5 wt%) with MFnetwork. The compression is conducted upto 50% of distortion.

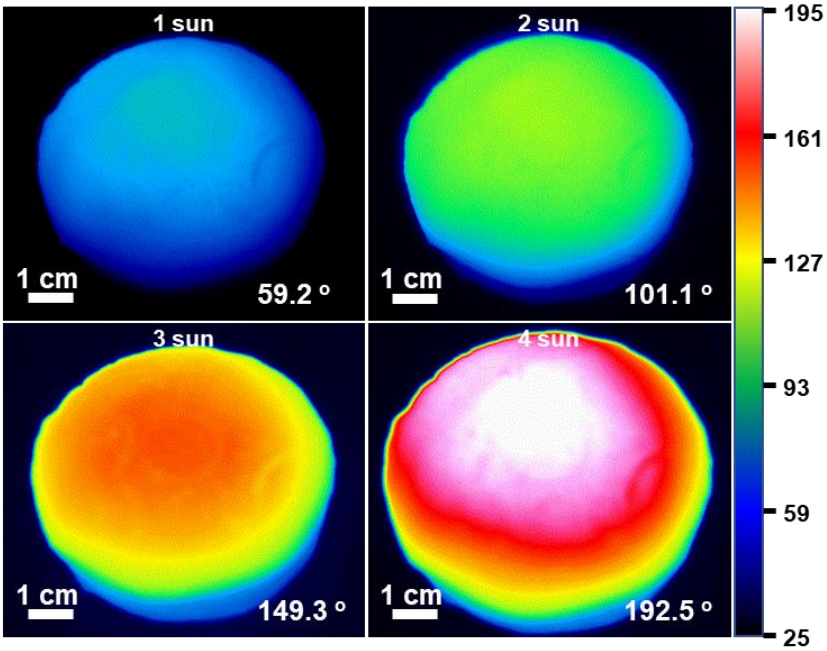

Figure S14. IR images of dried hydrogel under solar irradiation with increasing energy concentration from 1 to 4 sun.

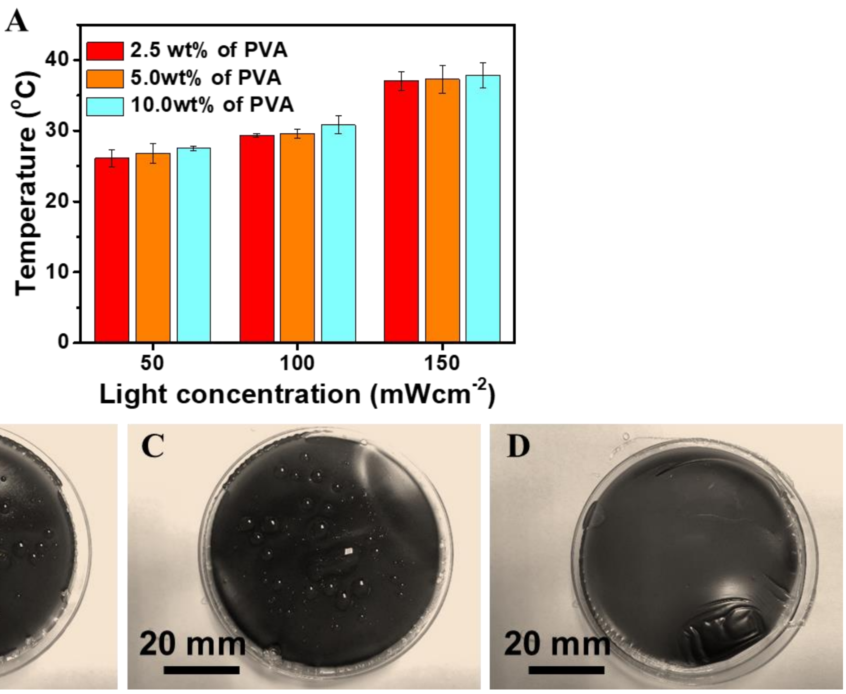

Figure S15. Photothermal properties of PVA-MF foam-rGO hydrogel. (A) Maximum temperature of the hydrogel surface under solar irradiation with various energy concentration. (B-D) Digital images of PVA-MF foam-rGO hydrogel with increasing PVA content from 2.5wt%, 5.0wt% to 10.0wt%, respectively.

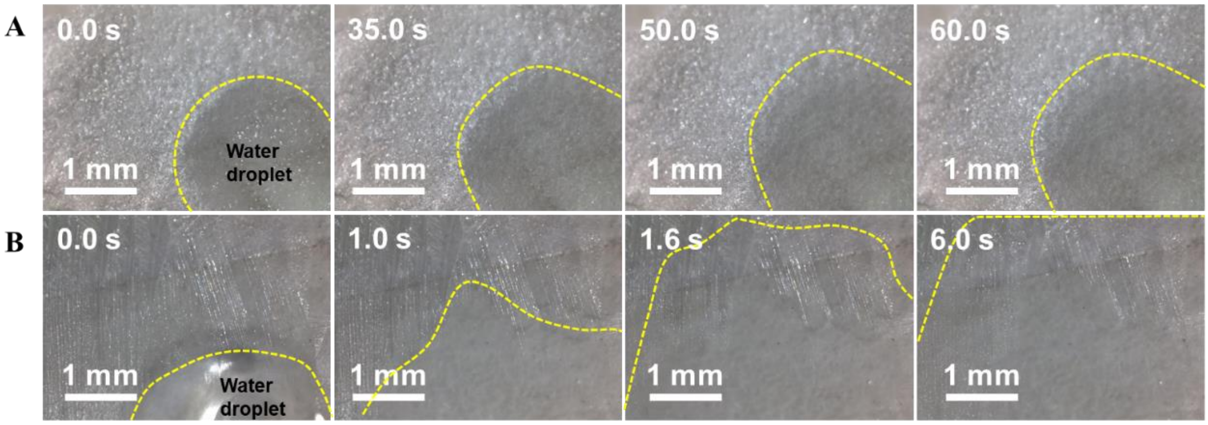

Figure S16. The effect of directional acetylated chitosan channel in the water transportation. (A) Porous acetylated chitosan without channel. (B) Acetylated chitosan with directional channels.

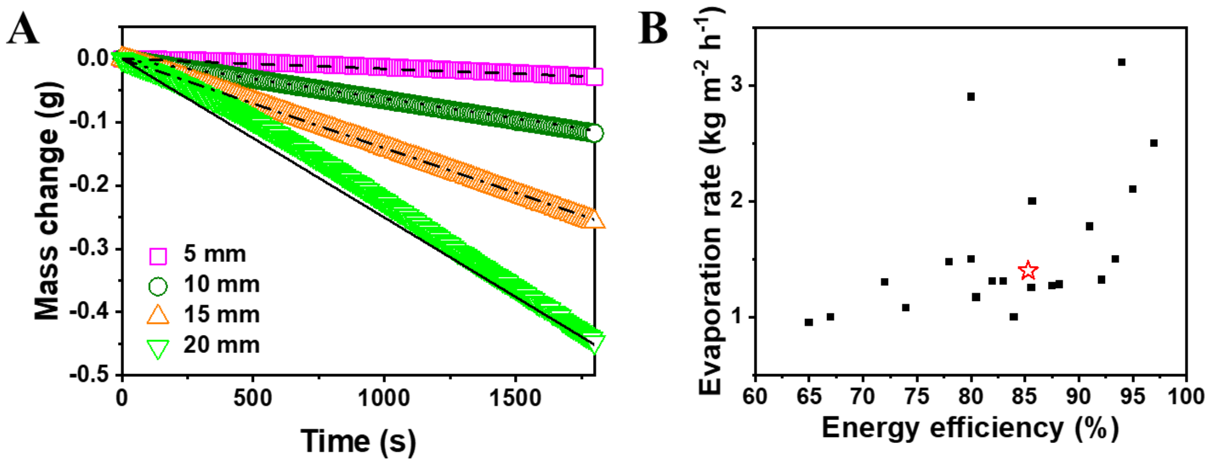

Figure S17. A, Mass change as a function of time in tubes of different diameters. B, omparison of solar steam generation rate and energy efficiency obtained by previously reported materials under one sun irradiation.[1-22]

Compared with previous reported evaporation generators, including wood, rGO based materials and polymer based materials, this trunk-leaf system shows a high solar thermal conversion efficiency calculated by the same data processing procedures (Figure S17B). It should be noted that since those counterparts do not demand anti-gravity water transport and distribution process, our system has extremely high efficiency. This biomimetic device will have potential to perform remote water harvesting and purification. Additionally, we also implemented water evaporation by tunning the number of trunks and the thickness of leaves, which allows for a rate-controllable clean water generation by choosing a proper device.

Supplementary note 1 – Estimates of the rising height and flow rate

The driven force results from the capillary force, $2\sigma\cos\theta/r$, denoting σ the surface tension of the liquid-vapor interface, θ the contact angle and r the characteristic radius of the pores of the pores in the microchannels of the silicone tube.

During the transport, the drag force is caused by the viscosity of the fluid in motion, i.e., $F_{drag} = \varepsilon\mu HU/K$, in which ε is the material porosity, K the permeability of the material, μ the viscosity of water, H the rising height, U the corresponding velocity and $U = \mathrm{d}H/\mathrm{d}t$. Since our tubes are quite long and much larger than the capillary length,[23] the effect of gravity has to be included. On the basis of the Lucas-Washburn equation,[24, 25] the balance of the capillary rise could be built as follows

$$\frac{2\sigma\cos\theta}{r} = \frac{\varepsilon\mu}{K} H \frac{\mathrm{d}H}{\mathrm{d}t} + \rho g H , \tag{S1}$$

in which ρ and g are the density of water and the acceleration of gravity. For convenience, by using $a = (K/\varepsilon\mu)(2\sigma\cos\theta/r)$ and $b = \rho g(K/\varepsilon\mu)$, we rewrite Eq. (S1) into

$$a = H\frac{\mathrm{d}H}{\mathrm{d}t} + bH . \tag{S2}$$

Taking into consideration the boundary conditions, i.e. $H|_{t=0} = 0$, we get the analytical solutions

$$U = \frac{\mathrm{d}H}{\mathrm{d}t} = \frac{a}{H} - b , \tag{S3}$$

$$t = -\frac{H}{b} - \frac{a}{b^2}\ln\left(1 - \frac{bH}{a}\right). \tag{S4}$$

The two unknown parameters a and b represent the material properties of the porous materials, and by employing the method of least-squares on the experimental data, we can determine their values. Moreover, and the amount of the mass flow $Q_F(t)$ and the mass flow rate $q_F(t)$could be estimated as follows

$$Q_F(t) = \pi R^2 \varepsilon \rho H(t), \tag{S5}$$

$$q_F(t) = \frac{dQ_F(t)}{dt} = \pi R^2 \varepsilon \rho \frac{dH(t)}{dt} = \pi R^2 \varepsilon \rho \cdot U = \pi R^2 \varepsilon \rho \left(\frac{a}{H} - b \right), \tag{S6}$$

in which R is the radius of the capillary tube.

From Eq. (S3)(S4), we get the following conclusions: (i) When $t_{max} \to \infty$, we could obtain $(1 - bH_{max}/a) = 0$ and then get the maximum rising height $H_{max} = a/b = 2\sigma\cos\theta/(\rho g r)$, which is controlled by the characteristic size of the porous of the materials; (ii) Meanwhile, from Eq. (S3), we can see that $t_{max} \to \infty$ and $(1 - bH_{max}/a) = 0$ also means $U|_{t max \to \infty} = 0$ and the flow finally stops, which is reasonable. However, in this case, the time needed is infinite, which is impossible in the real case, and it is the reason why we could only take experiments in a finite timespan; (iii) Since in our theoretical framework, we write the individual material properties (i.e. K, ε, μ, σ, θ, r, ρ and g) into the two parameters a and b, based on the experimental data and by employing the least square method, we can easily determine their values, which is much more convenient than measurements of the individual material properties separately; (iv) Even though our silicon tube is quite long and the effect of gravity cannot be ignored, the drag force cause by the gravity is smaller compared with the capillary force or the viscous force. In other words, the balance of Eq. (S1) is mainly a result of the competition between the capillary force and the viscous force. In such a case, if we just consider the capillary force and the viscous force (i.e. letting$b \to 0$), the solution of Eq. (S1) and Eq. (S4) will degrade to $H = (2at)^{1/2}$, this is the reason why we obtain a 1/2 power scaling $H \sim t^{1/2}$ in Figure 3B. In FigureS18B and Figure 3B, the fitted parameters are:$a = 4.20\times10^{-6}$ m^2 s^{-1} and $b = 2.789\times10^{-5}$ m s^{-1}, $a = 1.15\times10^{-5}$ m^2 s^{-1} and $b = 5.077\times10^{-5}$ m s^{-1}, $a = 2.0\times10^{-5}$ m^2/s and $b = 6.64\times10^{-5}$ m s^{-1}, $a = 3.0\times10^{-5}$ m^2/s and $b = 8.079\times10^{-5}$ m s^{-1}, and $a = 0.235\times10^{-6}$ m^2/s and $b = 5.050\times10^{-6}$ m s^{-1}for the tubes with the concentration of chitosan 0.5%, 1.0%, 1.5%, 2.0% and the disordered, respectively.

Moreover, based on the fitted values of the coefficients a and b in Eq. (S4), we could estimate the maximum height H_{max} of the capillary rise. For the tubes with the chitosan concentration ranges from 0.5% to 2.0%, H_{max} could reach values in the range[50mm, 400 mm]. Here, we only pursue an estimate of the order of H_{max}, a much more precise estimate of H_{max} needs to precisely determine the value of a and b, which needs more experimental data and a much

more time span to let the rising height reach a sufficient value, which is not very realizable in practical experiments considering we have already carried out experiments about 10^5 s. The data in FigureS18 and Figure 3B suggests by employing our method, we could potentially realize a quite high capillary rise.

On the basis of Eqs. (S5), (S6) and the obtained value of *a* and *b*, we could estimate the amount of the mass flow Q_F with time, as well as the mass flow rate in the capillary rise, as shown in Figure S18. Even though the mass flow rate is must faster than the evaporation rate (see Figure 2D), the very fast flow only happens in the initial stage of the capillary rise and it will suddenly stop when the front location of water meets the upper boundary of the tube. After this stage, the loss of water to the airimmediately starts and lasts due to the sunlight driven evaporation through the leaf, the capillary rise in the tube just guarantees a continuous transport of water from the root to the leaf to compensate the water loss due to the evaporation.

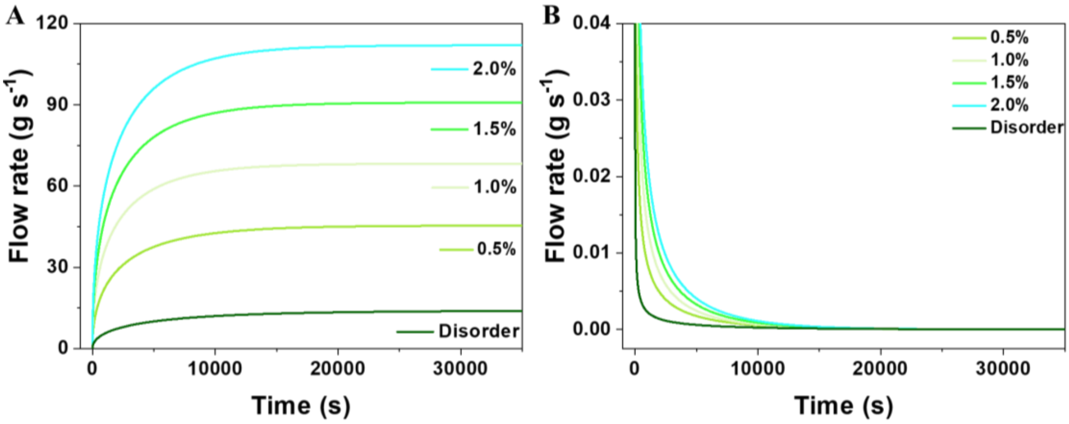

Figure S18. Values of mass flow and flow rate with time. The instantaneous mass flow (A) and the mass flow rate (B) as a result of the capillary rise in the tubes in the initial stage, respectively. The numbers 05.%, 1.0%, 1.5% and 2% represent the solution concentration of chitosan used to build up the microchannels in the trunk, and these values are related to different characteristic diameters of the microchannels, as shown in Figure S3.

Supplementary note 2 – Estimates of the evaporation rate

In the view of thermodynamics, the heat transfer q_T through the liquid-vapor interface could be written as[26]

$$q_T = \frac{dQ_T}{dt} = L\frac{dm}{dt} \quad \text{(S7)}$$

$$q_{\mathrm{T}} = \frac{\mathrm{d}Q_{\mathrm{T}}}{\mathrm{d}t} = \Delta T \cdot h_{\mathrm{i}} \cdot A \qquad \text{(S8)}$$

in which Q_{T} is the energy release during the phase change process (i.e., from liquid to vapor), t is the time, L is the latent heat of vaporization of water, ΔT is the temperature difference between water and vapor, h_{i} is the interfacial heat transfer coefficient, and A is the cross-sectional area of the tube.

A combination of Eq. (S7) and (S8) leads to

$$\frac{\mathrm{d}m}{\mathrm{d}t} = \frac{\Delta T \cdot h_{\mathrm{i}} \cdot A}{L}. \qquad \text{(S9)}$$

In our experiments, ΔT, h_{i}, A and L are constant, so we get $m = (\Delta T h_{\mathrm{i}} A/L)t$. For water in our working condition, we use $L = 2.256\times10^{6}$ J kg^{-1}, $h_{\mathrm{i}} = 180$ W K^{-1} m^{-2} (we consider the case of evaporation of free convection in the environment). The cross-sectional area A can be directly calculated from the tube with given diameters. In our experiments under solar irradiation of 1 sun, the temperature difference is about $\Delta T = 10$ °C. From Figure 3C in the main paper, we can see Eq. (S9) follows the experimental data very well.

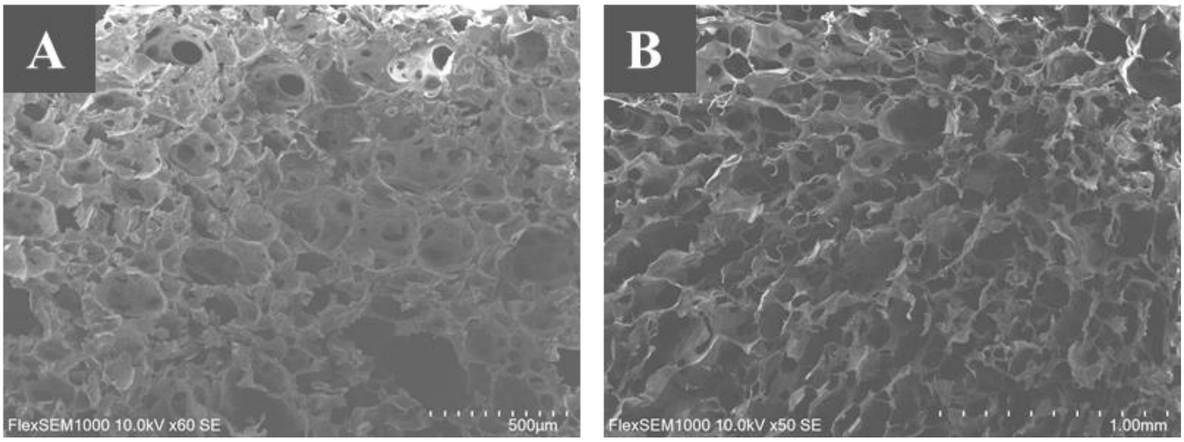

FigureS19. SEM images of the porous MF foam through top-view (A) and parallel cutting (B).

Table S1. Physicochemical properties of employed organic solvent.

Sample	BP (°C)	Saturated vapor pressure (kPa)	H_L (kJ kg^{-1})	Surface tension (mNm^{-1})	viscosity Pa s-1 (20 °C)	Density (gmL^{-1})
acetone	56.5	24 (20 °C)	501	18.8	0.316	0.80
methanol	64.7	12.3 (20 °C)	1102.4	18.8	0.5945	0.79
tetrahydrofuran	66	19.3 (20 °C)	410	28.8	0.55	0.89
ethyl acetate	77.2	10.1 (20 °C)	362	23.5	0.449	0.90
acetonitrile	81.6	13.33 (27 °C)	810	22.7	0.348	0.79
ethanol	78.3	5.8 (20 °C)	850	22.3	0.5945	0.79
Water	100.0	2.34 (20 °C)	2270	72.75	1.00	1.00
methylbenzene	110.6	3.8 (25 °C)	412.5	28.8	0.5866	0.87
chlorobenzene	131.7	1.17 (20 °C)	324.9	33.0	0.799	1.11
dimethyl sulfoxide	189.0	0.049 (20 °C)	677.3	43.6	1.1	1.10
N-Methyl pyrrolidone	204.0	0.53 (60 °C)	439.5	33.7	1.65	1.03

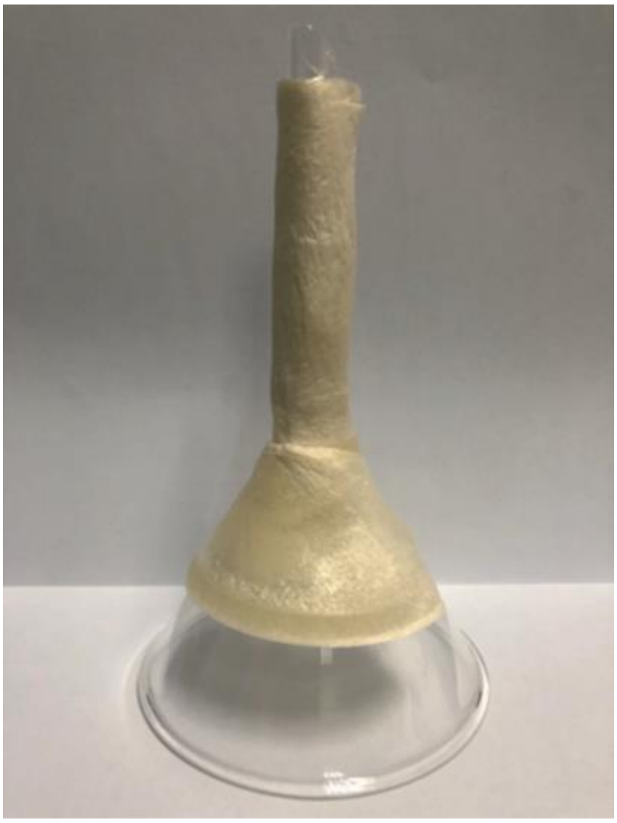

FigureS20. Digital camera image of a funnel coated with porous acetylated chitosan on its outside surfaceto improve its contact with organic solvents for solar steam condensation.

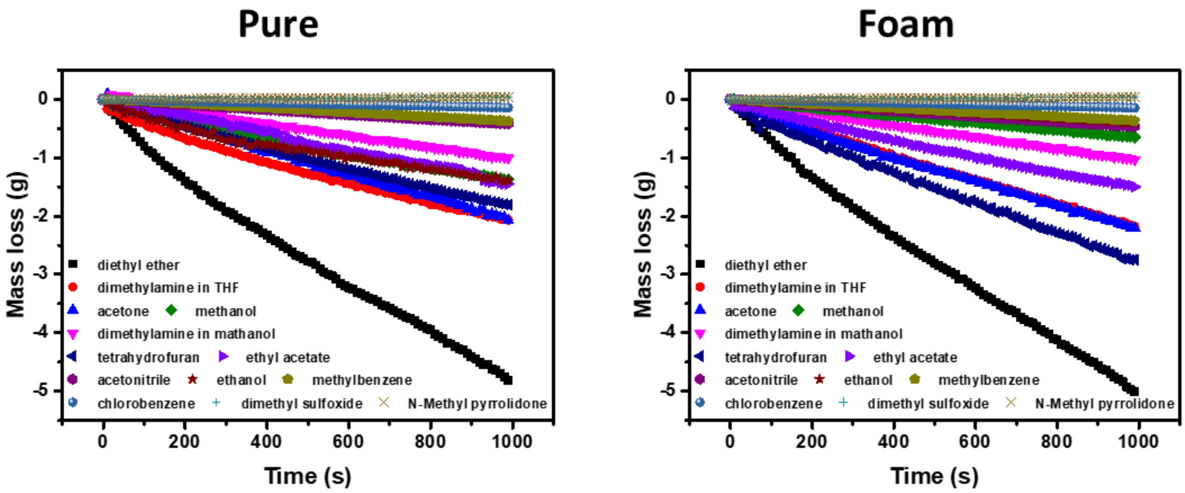

Figure S21. Real-time monitoring of liquids evaporation without (on the left) and with (on the right) the porous acetylated chitosan foam on the outside surface of the glass funnel.

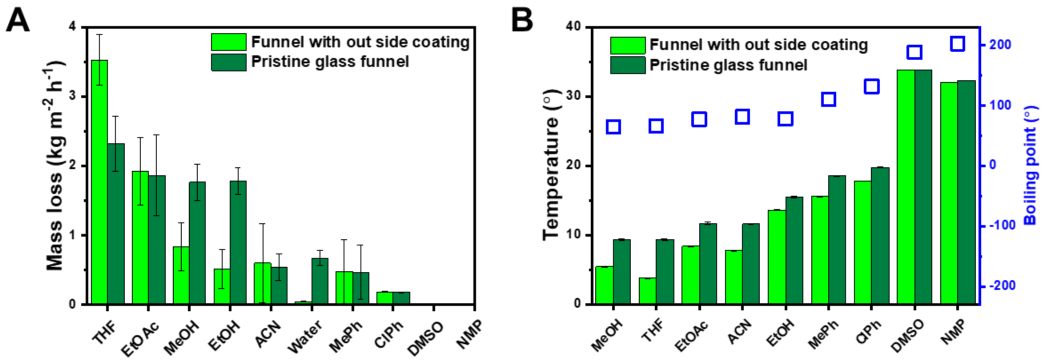

Figure 22. Evaporation cooling using organic solvents. (A) Mass change of liquids with and without chitin foam. (B) The evaporative cooling ability of various liquid and the corresponding boiling temperature. The temperature was recorded in the acetylated chitin foam filled with organic liquids and pure organic liquids, respectively.

References

1. K. Bae, G. Kang, S. K. Cho, W. Park, K. Kim and W. J. Padilla, *Nat. Commun.*, 2015, **6**, 10103.
2. G. Wang, Y. Fu, A. Guo, T. Mei, J. Wang, J. Li and X. Wang, *Chem. Mater.*, 2017, **29**, 5629-5635.
3. C. Li, D. Jiang, B. Huo, M. Ding, C. Huang, D. Jia, H. Li, C.-Y. Liu and J. Liu, *Nano Energy*, 2019, **60**, 841-849.
4. M. Zhu, Y. Li, F. Chen, X. Zhu, J. Dai, Y. Li, Z. Yang, X. Yan, J. Song, Y. Wang, E. Hitz, W. Luo, M. Lu, B. Yang and L. Hu, 2018, **8**, 1701028.
5. R. Li, L. Zhang, L. Shi and P. Wang, *ACS Nano*, 2017, **11**, 3752-3759.
6. H. Liu, C. Chen, G. Chen, Y. Kuang, X. Zhao, J. Song, C. Jia, X. Xu, E. Hitz, H. Xie, S. Wang, F. Jiang, T. Li, Y. Li, A. Gong, R. Yang, S. Das and L. Hu, 2018, **8**, 1701616.
7. Q. Chen, Z. Pei, Y. Xu, Z. Li, Y. Yang, Y. Wei and Y. Ji, *Chemical Science*, 2018, **9**, 623-628.
8. Y. Li, T. Gao, Z. Yang, C. Chen, Y. Kuang, J. Song, C. Jia, E. M. Hitz, B. Yang and L. Hu, *Nano Energy*, 2017, **41**, 201-209.
9. Y. Li, T. Gao, Z. Yang, C. Chen, W. Luo, J. Song, E. Hitz, C. Jia, Y. Zhou, B. Liu, B. Yang and L. Hu, 2017, **29**, 1700981.
10. Z. Liu, H. Song, D. Ji, C. Li, A. Cheney, Y. Liu, N. Zhang, X. Zeng, B. Chen, J. Gao, Y. Li, X. Liu, D. Aga, S. Jiang, Z. Yu and Q. Gan, 2017, **1**, 1600003.
11. W. Xu, X. Hu, S. Zhuang, Y. Wang, X. Li, L. Zhou, S. Zhu and J. Zhu, 2018, **8**, 1702884.
12. Y. Wang, L. Zhang and P. Wang, *ACS Sustainable Chemistry & Engineering*, 2016, **4**, 1223-1230.
13. J. Wang, Y. Li, L. Deng, N. Wei, Y. Weng, S. Dong, D. Qi, J. Qiu, X. Chen and T. Wu, 2017, **29**, 1603730.
14. L. Shi, Y. Wang, L. Zhang and P. Wang, *Journal of Materials Chemistry A*, 2017, **5**, 16212-16219.
15. N. Xu, X. Hu, W. Xu, X. Li, L. Zhou, S. Zhu and J. Zhu, 2017, **29**, 1606762.
16. H. Ren, M. Tang, B. Guan, K. Wang, J. Yang, F. Wang, M. Wang, J. Shan, Z. Chen, D. Wei, H. Peng and Z. Liu, 2017, **29**, 1702590.
17. Y. Ito, Y. Tanabe, J. Han, T. Fujita, K. Tanigaki and M. Chen, 2015, **27**, 4302-4307.
18. P. Zhang, Q. Liao, T. Zhang, H. Cheng, Y. Huang, C. Yang, C. Li, L. Jiang and L. Qu, *Nano Energy*, 2018, **46**, 415-422.
19. P. Zhang, Q. Liao, H. Yao, H. Cheng, Y. Huang, C. Yang, L. Jiang and L. Qu, *Journal of Materials Chemistry A*, 2018, **6**, 15303-15309.
20. X. Zhou, F. Zhao, Y. Guo, Y. Zhang and G. Yu, *Energy & Environmental Science*, 2018, **11**, 1985-1992.
21. F. Zhao, X. Zhou, Y. Shi, X. Qian, M. Alexander, X. Zhao, S. Mendez, R. Yang, L. Qu and G. Yu, *Nat. Nanotechnol.*, 2018, **13**, 489-495.
22. Q.-F. Guan, Z.-M. Han, Z.-C. Ling, H.-B. Yang and S.-H. Yu, *Nano Lett.*, 2020, DOI: 10.1021/acs.nanolett.0c01088.
23. P.-G. De Gennes, F. Brochard-Wyart and D. Quéré, *Capillarity and wetting phenomena: drops, bubbles, pearls, waves*, Springer Science & Business Media, 2013.
24. M. Lago and M. Araujo, *J. Colloid Interface Sci.*, 2001, **234**, 35-43.
25. J. Hyväluoma, P. Raiskinmäki, A. Jäsberg, A. Koponen, M. Kataja and J. Timonen, *Phys. Rev. E*, 2006, **73**, 036705.

26. L. G. Leal, *Advanced transport phenomena: fluid mechanics and convective transport processes*, Cambridge University Press, 2007.
